# Ovarian Cancer Management in the Oldest Old: Improving Outcomes and Tailoring Treatments

**DOI:** 10.14336/AD.2017.0607

**Published:** 2017-10-01

**Authors:** Lucia Tortorella, Giuseppe Vizzielli, Domenico Fusco, William C. Cho, Roberto Bernabei, Giovanni Scambia, Giuseppe Colloca

**Affiliations:** ^1^Division of Gynecologic Oncology, Catholic University of the Sacred Heart, Rome, Italy.; ^2^Geriartic Department, Fondazione Policlinico A.Gemelli, Rome, Italy; ^3^Department of Clinical Oncology, Queen Elizabeth Hospital, Kowloon, Hong Kong

**Keywords:** ovarian cancer, elderly, frailty

## Abstract

Ovarian cancer is the most common cause of death from gynecological cancers in developed countries. It is a common disease of older women at or above 63 years upon diagnosis. Thanks to advance in new treatments, mortality from ovarian cancer has declined in developed countries in the last decade. This decline in mortality rate is unevenly distributed across the age-spectrum. While mortality in younger women has decreased 21.7%, for elderly women it has declined only 2.2%. Even if ovarian cancer is clearly a disease of the elderly, older women are underrepresented in clinical trials, and scant evidence exists for the treatment of women older than 80 years. Moreover, older women are frequently undertreated, receive less chemotherapy and less combination of surgery and chemotherapy, despite the fact that this is considered the optimal treatment modality. This may be mainly due to the lack of evidence and physician’s confidence in the management of elderly women with ovarian cancer. In this review, we focus on the management of older women with ovarian cancer, considering geriatric features tied to this population.

Ovarian cancer is the third most common and the first cause of death from gynecologic cancer. The incidence of this disease rises with advancing age, peaking in the seventh decade of life. In U.S. 22,280 new cases are projected in 2016, 44% of whom occur in women over the age of 65 (1-2).

Unfortunately, survival outcomes are poor in older patients, as shown by a study on 28,165 patients. Across all stages, very young women had a significant survival advantage over the young and older groups with 5-year disease-specific survival estimates at 78.8% vs. 58.8% and 35.3%, respectively (P=0.001) (3).

*Wright* et al, conducted a study on 49, 932 women with ovarian cancer diagnosed from 1975 to 2011, and the results showed that for women with stage III and IV tumors, excess mortality is greater for older women at 1 and 5 years (1-y excess HR: 2.71 for 80 or older relative to 50-59; 5-y excess HR: 6.4 for 80 or older relative to 50-59). Among all stages, survival decreased with increasing age and with time since diagnosis. The decrease in relative survival was more pronounced for women with advanced-stage tumors. Excess hazard ratios for death at 1-year and 5-years was higher for old compared to young patients, while at 10-years age did not affect mortality rate (10 years HR: 0.91 CI 99% 0.50-1.62 for young patients and HR: 0.87 CI 99% 0.05-14.48 for older patients) (4).

The reason of poorer prognosis of older patients is not well defined; a number of factors may influence the outcome. It has been demonstrated that increasing age is associated with more advanced stage at diagnosis and potentially with adverse tumor biology.

However, the EUROCARE study showed that the difference in survival between elderly and middle-aged patients was much greater at 1-year after diagnosis than that at 5-years underlining that elderly patients surviving the first year have a prognosis similar to middle-aged patients (5).

Thus, while mortality and incidence of ovarian cancer increase with patients’ aging, elderly patients themselves express a strong wish to receive radical and curative treatment (6).

In clinical practice, the assessment of oldest old patients should not be based only on chronological age, but multidimensional evaluation should be able to identify patients that could benefit from a more intensive treatment.

## Geriatric aspects of patients with ovarian cancer

Older women with cancer present with some medical and physiological conditions that deserve special attention in planning treatment for ovarian cancer. Multimorbidity, disability and polypharmacotherapy have been shown to predict adverse outcomes in cancer patients. In particular comorbidity was associated with mortality and surgical complications in older women with ovarian cancer (7,8).

Aging is associated with increased prevalence of chronic disease, a progressive decrease of organ function and, as a consequence, it is possible to observe pharmacodynamic and pharmacokinetic changes of drugs in terms of solubility, therapeutic window, distribution volume (i.e. hydrophilic vs. lipophilic drug), prevalence of adverse drug reactions.

The physiologic changes of aging in the organ systems do not produce any evident clinical problem if those lie in a sort of a homeostatic scenario, but under stress (surgical intervention, radiotherapy, chemotherapy etc.) there may be an inadequate functional reserve (frail patients), and it’s easier to undergo some adverse drug reactions or treatment complications (9).

Frailty is a geriatric syndrome, characterized by reduced homeostatic reserve, i.e. reduced capacity of coping with acute and environmental stressors, posing the individual to higher risk of negative health-related outcomes (falls, disability, institutionalization and death) (10,11). Even though it is strictly related to disability and multimorbidity, frailty is a distinct condition. It is highly prevalent in older cancer patients and clearly associated with adverse outcomes (12). Ovarian cancer and its complex multimodality treatment (surgery and chemotherapy) can constitute significant stressors, making possible for a frail old woman the transition from a condition of self-sufficiency to overt disability. This could lead to institutionalization and eventually to death. Many definitions of frailty have been proposed. The frailty phenotype identified by Fried and colleagues (13) is characterized by a combination of unintentional weight loss, self-reported fatigue, diminished physical activity, reduced strength and gait speed. A person is identified as frail by the combination of three or more of these markers, while the presence of only one or two of them identify the person as pre-frail. Courtney-Brooks et al applied the frailty phenotype model in a small group of gynecologic oncology patients showing that frailty is a possible predictor for postoperative morbidity (14).

From a clinical perspective, physical frailty is easily revealed by the occurrence of mobility disability (15,16), which is strictly related to the loss of function of skeletal muscle. Mobility disability offers an opportunity to objectively measure frailty in a simple way, making easier its recognition in many different clinical settings. Simple performance tests can be included in the clinical evaluation of elderly cancer patients. Gate speed, Timed-Up-and-Go Test (TUG) and Short Physical Performance Battery have been extensively used to assess physical performance in cancer patients in clinical studies (17,18). In particular, their usefulness has been demonstrated in gynecological malignancies, where SPPB and usual gate speed were significantly associated with mortality (19).

Mobility disability and frailty are strictly entangled with the occurrence of sarcopenia, a condition of reduced muscle mass and function, that characterizes older age (20). The presence of low muscle mass has been frequently reported in patients with cancer and constantly related to adverse outcomes (either reduced disease free survival, recurrence or overall survival) (21). In particular, Kumar et al. recently reported a high frequency of sarcopenia in patients with advanced epithelial ovarian cancer (AEOC) undergoing primary debulking surgery (44.6% of their sample), that was associated with reduced survival (22). Moreover, Rutten et al described a higher mortality rate in women with AEOC undergoing neoadjuvant chemotherapy who lost muscle mass. Interestingly, the occurrence of low muscle mass at baseline was not predictive of adverse outcomes, underlying the importance of checking for sarcopenia through the entire course of treatment (23).

Comprehensive geriatric assessment (CGA) gives the opportunity to identify problems in elderly patients with cancer, that are commonly missed at routine clinical evaluation (24). It facilitates the identification vulnerable older adults, predicting treatment-related toxicities and adverse outcomes. This allows the subsequently tailoring of cancer treatment to the specific needs of frail patients. It has been recommended that some domains be routinely evaluated in a CGA: functional status, comorbidity, cognition, mental health status, fatigue, social status and support, nutrition, and the presence of geriatric syndromes (25).

Many different tools of CGA have been developed. The Preoperative Assessment of Cancer in the Elderly (PACE) has been designed to be used in the pre-surgical evaluation of elderly patients. In this model, IADL, fatigue, performance status, were all predictive surgical complications, while ADL, IADL and performance status were predictive of extended hospital stay (26). A modification of the PACE tool is under evaluation for the pre-surgical assessment of older women with ovarian cancer (27).

Other instruments have been tested to assist the evaluation of older patients undergoing chemotherapy, in order to prevent toxicities. The Cancer and Aging Research Group (CARG) investigators identified a toxicity score based on CGA that predicted the risk of toxicities better than performance status (28). In their model, older age, cancer type, chemotherapy variables (standard dosing and polychemotherapy), anemia, renal insufficiency, decreased hearing, falls, dependency in IADL, limitations in walking ability, decreased social activity, were all associated with grades 3-5 chemotherapy toxicities.

Specific instruments have been designed in the field of gynecologic oncology to predict the risk of adverse reactions from chemotherapy in older women with AEOC. The geriatric vulnerability score (GVS) has been developed by the French Group d’Investigateurs Nationaux pour l’Etude des Cancers Ovariens (GINECO) (29). It is the sum of the following features: albuminaemia <35 g/l; ADL score <6; IADL score <25; lymphopenia <1 G/l; and HADS >14. Patients with a GVS score ≥3 showed reduced OS, treatment completion, adverse events and unplanned hospital admissions rates. The GVS score is currently being tested in a clinical trial (27).

At present, no single instrument has been proven to be clearly superior to the others in the identification of frailty and prediction of adverse outcomes of cancer treatments. Also, differences in health care systems and resources make difficult the adoption of a single instrument. It seems reasonable to suggest that any institution chooses the tool that clinicians are more familiar with, within the ones that have been evaluated in clinical studies.

## Ovarian cancer management

### Surgery

Optimal treatment of advanced epithelial ovarian cancer (AEOC) consists of primary debulking surgery (PDS) followed by platinum-based chemotherapy. Residual tumor after surgery is one of the most important prognostic factors for survival. Bristow et al demonstrated that each 10% increase in percent maximal cytoreductive surgery is associated with a 5.5% increase in median survival time (30). Increasing age is associated with lower rates of cytoreductive surgery and lower rates of optimal cytoreduction, possibly due to a higher risk of postoperative morbidity and mortality. The ability to optimally cytoreduce elderly patients has been matter of debate in the recent literature (31-34).

Langstraat et al (31) demonstrated that age is an independent predictor of OS. OS decreased with increasing age (3.4 vs 1.6 years respectively for patients age 65-69 and > 80).

Older patients experienced high rates of surgical morbidity and mortality and were more frequently unable to receive chemotherapy. In multivariate analysis age was an independent predictor for both poor surgical outcome and OS. Moreover, they observed that median survival was similar, regardless of age, in patients optimally cytoreduced (RT=0), while age impacted significantly on survival in patients with RT>1 cm (0.5 vs. 2 years respectively for patients age 65-69 and > 80 not optimally cytoreduced). Older patients were more likely to undergo less complex surgery with a higher proportion of RT>1 cm. The challenge is to identify elderly patients who benefit from complete multimodal treatment, whose outcome is equivalent to that of younger women.

A retrospective analysis has been conducted in three tertiary cancer centers in the USA to identify independent predictors of poor outcome in patients whit AEOC (32). The feasibility of complete cytoreduction is related not only to tumor characteristics (stage and distribution), but also to a patient’s ability to tolerate a complex surgical procedure (age, performance status, nutritional status). They concluded that age > 75, ASA score > 3, preoperative albumin level and stage defined a high-risk group. The median OS was 17 months in this group, compared to 40.2 months in the overall study population with stage III and IV AEOC.

In contrast, a study (33) found that younger (under 70 years) and older (over 70 years) women were equally debulked reporting the same percent of morbidity with no significant difference in survival between the 2 cohorts. The conclusion was that aggressive surgical cytoreduction for ovarian cancer is safe and feasible in elderly patients. Patients should not be offered less aggressive treatment based on age alone: carefully selected elderly patients tolerate surgical cytoreduction remarkably well with a complication rate similar to that of younger women. Fanfani et al (34) also demonstrated that elderly (65-75 years) and very elderly (> 75 years) patients might tolerate radical and ultra-radical surgery without an increase of morbidity and with clinical outcomes similar to those reported in younger cases.

In particular, patients undergoing complete/optimal cytoreduction at PDS showed better prognosis compared with interval debulking surgery (IDS), except for very elderly patients in whom no significant difference was found between PDS and IDS. Maximal surgical effort should be considered the gold standard in the surgical management of EAOC older patients, avoiding selection based only on age.

In conclusion, despite the correlation between age and risk of surgical morbidity, several studies emphasize that elderly patients are able to tolerate a standard, aggressive approach when adequately selected (6, 35).

On the other hand neoadjuvant chemotherapy (NACT) with interval debulking surgery has been shown to be associated with higher rates of optimal cytoreduction, lower perioperative morbidity and mortality rates and similar outcomes than primary debulking surgery (36). Although it is a debated topic, the use of NACT could be preferable in frail patients.

A study conducted on 9,587 elderly patients with stage II-IV ovarian cancer showed that the use of primary surgery decreased from 63.2% in 1991 to 49.5% in 2007, while primary chemotherapy increased from 19.7% in 1991 to 31.8% in 2007 (*p*<0.0001) and there was no difference in terms of survival (37).

## Chemotherapy

Elderly patients often do not receive standard chemotherapic treatments compared to younger patients because of the presence of multimorbidity, poor physical or cognitive performance and the risk of mortality. Common chemotherapy toxicities in the oldest old are grade 3-4 hematologic and gastroenterologic toxicities and grade 3-4 neutropenia.

Furthermore, Janda et al. demonstrated, on a cohort of 3994 women diagnosed with stage III or IV ovarian cancer, that despite age, FIGO stage, presence of comorbidities are usually considered risk factors of death within the first year from diagnosis, the strongest risk factor for death was not receiving the standard treatment (combination of chemotherapy and surgery) (6).

A retrospective study on 184 elderly patients showed that dose-delay was associated with a decrease in OS, that remained significant after adjustment for age, stage, residual disease and number of chemotherapy cycles received (38). Another retrospective analysis showed that reduced-dose carboplatin/paclitaxel may be better tolerated but equally effective as the standard regimen in elderly ovarian cancer patients with AEOC. There were no differences in Progression Free Survival (PFS) or Overall Survival (OS) between cohorts (OS 41 months and 44 months respectively in the lower dose and in the regular dose group, p=0.4) (39).

A phase III study on 779 patients evaluated first line chemotherapy with carboplatin/paclitaxel versus cisplatin/paclitaxel following cytoreductive surgery for advanced ovarian cancer. Authors concluded that combination chemotherapy was tolerable in an older population but early discontinuation of therapy was double in patients over the age of 70 compared to younger patients (40).

MITO-5 trial is a phase II study conducted to evaluate the tolerability of a weekly schedule of carboplatin (2 AUC) and paclitaxel (60 mg/mq) in patients aged > 70 years. They concluded that this regimen is safe and well tolerated with 65% of those patients receiving all six cycles (41). MITO-7 is a multicentric, randomised, phase 3 study conducted in 67 institutions that showed that carboplatin and paclitaxel given once a week vs every 3 weeks did not significantly prolong PES regardless of age (> 70 years vs. <70 years) but it is associated with better quality of life and fewer toxic effects (42).

### Bevacizumab

Bevacizumab is a humanized vascular endothelial growth factor (VEGF)-neutralizing monoclonal antibody that inhibits tumor angiogenesis. Several phases 3 trials have shown that bevacizumab, in combination with chemotherapy, improves PFS in the first-line setting of advanced ovarian carcinoma and also in platinum sensitive and platinum-resistant relapses but there is a lack of clinical trials focusing on older patients.

GOG-218 is a phase III randomized trial about the use of bevacizumab in association with chemotherapy in first line ovarian cancer, in which bevacizumab prolongs the median PFS by about 4 months in all ranges of age (43). OCEANS is a randomized, phase III trial evaluating the addition of a biologic therapy to standard chemotherapy in platinum sensitive recurrent ovarian cancer. Also in this setting of patients the use of bevacizumab resulted in a statistically significant improvement in PFS among both patients aged above and below 65 years (44).

AURELIA study (45) showed that also in platinum-resistant ovarian cancer adding bevacizumab to standard single-agent chemotherapy (pegylated liposomal doxorubicin, weekly paclitaxel, or topotecan) improves PFS. Significant benefits from the addition of bevacizumab in terms of PFS and response rate were seen in both older and younger patients.

Beinse et al. (46) conducted a study among older ovarian cancer patients in order to assess the tolerance of bevacizumab and identify a subpopulation of patients with a high risk of severe adverse effects. The median age at first infusion of bevacizumab was 67.5 years. There was no difference in terms of arterial, venous thromboembolism, hemorrhage, and bowel perforation or fistula related to age. The incidence of severe hypertension was significantly higher in elderly patients than in younger ones (39% vs 17%, P = 0.017). There were no differences in terms of PFS and OS in all ages.

Preliminary results of the OTILIA study (a single arm study that would assess the safety and efficacy of this regimen in routine oncology practice), showed no evidence that age is associated with worse outcome (preliminary efficacy or tolerability) in patients receiving Bevacizumab for primary OC (median PFS= 22.6 months for age < 70 years, PFS = 20.2 months for age > 70) (47).

Bevacizumab is an active and tolerable front-line treatment option that should be considered irrespective of age.

### BRCA assessment and poly-ADP-ribose polymerase (PARP) inhibitors

One of the most significant risk factors for the development of ovarian cancer is a genetic mutation in *BRCA1* or *BRCA2*. About 28.6% of patients with a germline *BRCA* mutation were aged above 60 years at diagnosis. Although young age at diagnosis is a characteristic informative of germline *BRCA* mutation in patients with OC, around one out of four germline *BRCA* mutation carriers may be diagnosed with OC above 60 years of age (48).

PARP inhibitors have shown significant clinical activity in women with BRCA mutation. Olaparib is a PARP enzyme inhibitor that selectively kills tumor cells with an impaired homologous recombination DNA repair pathway while sparing normal cells. Ledermann et al. (49) showed that PFS was significantly longer with olaparib than with placebo (median, 8.4 months vs. 4.8 months, *p*<0.001) among patients with platinum-sensitive, relapsed, high-grade serous ovarian cancer. Median age was 58 years (range 21-89 years)

No data is available at present on the tolerability and efficacy of olaparib in the elderly populations, making difficult to provide specific recommendations. Moreover, there may be problems of compliance to the treatment due to the dosage of 16 capsules per day in patients that are likely to be taking multiple other medications.

## Hyperthermic Intraperitoneal Chemotherapy (HIPEC)

It has been demonstrated the survival improvement in patients affected by peritoneal malignancies (pseudomyxoma peritonei, colon cancer, peritoneal mesothelioma) treated with HIPEC and cytoreductive surgery. In advanced ovarian carcinoma, there are not prospective, randomized studies but the results of the use of HIPEC after cytoreduction in primary or recurrent ovarian cancer appears to be encouraging.

HIPEC and cytoreductive surgery are associated with high morbidity and mortality rates. The largest multicentric study on 795 patients reported rates of major complications of 24-31% and mortality of approximately 2% (50). Advanced age is associated with 30-day death and morbidity but few studies in literature include elderly patients. A retrospective multicentric study on 1,085 patients has been conducted in order to identify preoperative risk factors that would significantly increase the risk of death and serious morbidity. They demonstrated a linear increase in mortality and severe morbidity of 0.6% per year beginning at age of 50 years, passing from a rate of 32% at 50 years to a rate of 53% for patients > 80 years. Moreover, age >60 years remained an independent risk factor in multivariate analysis (OR 1.6, p=0.001). Among the older population, they were able to identify preoperative risk factors for morbidity and mortality. A score was developed on the basis of the number of risk factors that were significant in multivariate analysis (weight loss, hypoalbuminemia, performance of splenectomy, operative time, intraoperative blood transfusion, dirty wound) (51). Age alone does not preclude safe performance of HIPEC, but the accurate selection of patients is essential to prevent unwanted negative outcomes.

## Immunotherapy

The introduction of immunotherapy causes a paradigm shift in the treatment of a number of cancers. Presence of tumor infiltration lymphocytes and PDL1 expression have been reported to affect prognosis in ovarian cancer (50-52)

Some clinical studies have tested immunotherapies in EOC, usually in platinum-resistant cases. Both anti-PD-L1 (nivolumab, pembrolizumab, avelumab) and anti-CTLA4 (ipilimumab) have been tested in phase I or phase II clinical trials, with objective response rates about 15% and some stable response. Currentl, some phase III clinical trials are ongoing with avelumab (53,54).

The number of older patients recruited in clinical trials with immunotherapies for cancer is generally low, thus preventing from definitive conclusions about safety and tolerability of these agents (55).

Cancer vaccine represents another interesting treatment avenue. DNA engineered autologous whole cell therapy, gemogenovatucel-T (Vigil®), incorporating the rhGMCSF (recombinant human granulocyte-macrophage colony stimulating factor) cDNA and the bifunctional shRNA (short hairpin ribonucleic acid) targeting furin have been tested in a phase II trial, giving encouraging results (56).

## Conclusions

The number of elderly people diagnosed with cancer and living with cancer will grow over the coming decades due to longer life expectancy and increased survival, further highlighting the importance of research in the elderly in order to provide a culturally competent and rational management. Further research needs to be done to identify elderly patients who could benefit from active treatment, whereas treatment decisions based mainly on chronologic age should be avoided. The construction of an oncogeriatric team could improve the selection of high-risk patients ensuring tailored treatment.
